# Learning Periodic Patterns in ECG Signals Using TimesNet for Automated Cardiac Classification

**DOI:** 10.3390/biomedicines14061198

**Published:** 2026-05-26

**Authors:** Manjur Kolhar, Raisa Nazir Ahmed Kazi, Ahmed M. Al Rajeh

**Affiliations:** 1 Department of Health Information Management and Technology, College of Applied Medical Sciences, King Faisal University, Al-Ahsa 36362, Saudi Arabia; 2 Department of Respiratory Therapy, College of Applied Medical Sciences, King Faisal University, Al Ahasa 31982, Saudi Arabia; rnahmed@kfu.edu.sa (R.N.A.K.); amalrajeh@kfu.edu.sa (A.M.A.R.)

**Keywords:** atrial fibrillation, deep learning, electrocardiography, explainable artificial intelligence, myocardial infarction, premature ventricular contraction, ST-T abnormalities, temporal feature extraction

## Abstract

**Background/Objectives**: Although deep learning methods have achieved promising performance in recent years, comparatively less attention has been given to explicitly modeling periodic and multi-scale temporal dynamics for ECG-specific representation learning within TimesNet-based frameworks. In this work, we propose an ECG-specific TimesNet-based framework for multi-label classification of multi-lead ECG recordings that incorporates periodicity-aware temporal modeling. **Methods**: The proposed framework utilizes Fast Fourier Transform (FFT)-guided temporal decomposition to identify dominant frequency components and reshapes ECG sequences into period-aligned representations to better capture intra-period morphological patterns and inter-period rhythm dependencies. Multi-scale convolutional TimesBlocks are further employed to learn rhythm-aware and morphology-aware temporal representations. **Results**: The proposed framework was evaluated on the PTB-XL dataset using two experimental settings: Three-Class classification (NORM, AFIB, PVC) and Five-Class classification (NORM, AFIB, MI, PVC, STTC). Experiments were conducted using a one-vs-rest multi-label learning strategy with independent class probability estimation. The framework achieved mean one-vs-rest test AUC values of 0.956 and 0.913 for the Three-Class and Five-Class settings, respectively. Experimental results indicated that the reduced classification complexity in the Three-Class setting was associated with improved feature separability, more stable decision boundaries, and enhanced discriminative representation learning. Latent-space visualization using UMAP and PCA demonstrated clearer clustering in the Three-Class configuration, while gradient-based interpretability analysis highlighted physiologically relevant ECG waveform regions contributing to model predictions. In addition, computational profiling demonstrated practical feasibility with approximately 1.957 million trainable parameters, 13.14 GFLOPs computational complexity, 5.230 ms average inference latency per ECG recording, and a throughput of approximately 191 ECG recordings per second on GPU hardware. **Conclusions**: These findings suggest that periodicity-aware temporal modeling can improve ECGF representation learning while demonstrating practical potential for computationally efficient and interpretable automated ECG analysis applications.

## 1. Introduction

Deep learning models have recently achieved strong performance in extracting highly informative features from the raw ECG signal and therefore improved diagnostic accuracy for various cardiovascular diseases [1,2,3,4,5,6,7,8]. However, it is still quite challenging for regular deep learning structures to capture the underlying temporal dynamics and inherent periodic characteristics embedded in the ECG signal [9,10,11,12,13,14]. Recent studies are devoted to investigating the new models for dealing with the challenges in time-series data analysis by exploiting the time-series patterns in the data, such as periodicity and multi-scale correlations in the data sequences [15,16,17,18,19,20]. A new deep learning framework known as TimesNet [21] was recently introduced that can be utilized for general time-series analysis. A regular deep neural network processes the information strictly in the time domain. However, unlike the previous models, TimesNet also utilizes a frequency-aware embedding which discovers the most influential patterns or characteristics in the signal associated with the dominant frequency components through utilizing the Fast Fourier Transform (FFT). FFT is commonly used in ECG signal processing to analyze signal in frequency domain and to extract features of cardiac signal to support for automated diagnosis. By detecting these periodic structures, the model reshapes the time-series signal into a two-dimensional representation that enables more effective learning of temporal variations. By introducing the periodic transformation, the proposed framework enables learning of intra-period and inter-period temporal relationships for convolutional operations. Hence, the deep learning model can learn more complex temporal relationships.

The ECG signals are periodic in nature as the heartbeats produce the periodic cardiac cycles generated due to the electrical activity of the heart. Each cardiac cycle consists of characteristic waveform components. The P wave, the QRS complex and the T-wave are the major components of the waveform. The P wave is associated with the depolarization of the atria. The QRS complex is associated with the depolarization of the ventricles. The T-wave is associated with the repolarization of the ventricles. Many abnormalities in cardiac function are revealed through changes in the waveform patterns that are observed in the ECG signal. Therefore, it is essential to capture both the morphology of the individual beats as well as the relationships between them when classifying the cardiac function from the ECG signal. The proposed TimesNet-based framework leverages these properties through its periodicity-aware architecture that is well-suited for physiological signal processing.

In this study, a periodicity-aware TimesNet-based framework was designed for multi-label ECG classification using multi-lead ECG recordings to construct a TimesNet-based deep network called MultiLabel_ECG_Classification. Meanwhile, FFT-guided temporal decomposition and multi-scale temporal feature learning were combined to capture rhythm-related temporal dependencies and morphology-related waveform characteristics for different cardiac conditions.

This study explores how increasing the number of classes in a classification task influences the separability of the features learned, the decision boundary and the confidence of the classifier. The experimental analysis compares the performance of the proposed framework under Three-Class and Five-Class ECG classification settings while investigating the impact of classification complexity on the learned representation space. Our experimental results provide a detailed analysis of how increasing the number of classes affects the representational geometry of periodic physiological signals and the performance of periodicity-aware deep learning models. We further investigate how incorporating periodicity-aware temporal modeling together with visualization-based interpretation techniques may assist automated ECG analysis and support clinicians during diagnostic assessment.

To investigate the effect of classification complexity on classifier performance, we carry out experiments in two settings, namely, Three-Class and Five-Class. The Three-Class configuration is for rhythm-based classes of Normal Sinus Rhythm (NORM), atrial fibrillation (AFIB) and premature ventricular contraction (PVC). These classes are chosen as they are the most common arrhythmia and as they have distinct temporal characteristics that can be identified using the model. The Five-Class configuration is an extension of the classification problem with the addition of myocardial infarction (MI) and ST-T changes (STTCs). Although this gives a more comprehensive diagnostic test, it introduces a higher degree of difficulty due to the fact that some cardiac abnormalities have very similar morphological characteristics. Feature-space visualization techniques, including PCA and UMAP, were further utilized to investigate latent-space organization, class separability, and clustering behavior of ECG representations learned by the proposed framework.

Through the application of PCA or UMAP on the neural network features, we can get some insights into the way the ECGs are positioned within the feature space. Visualizing the ECGs and analyzing the clusters and the separability of different classes (cardiac conditions) can help us gain more information about the performance of the model. In addition, probability landscape visualizations give us more information about the variability of prediction confidence across the entire feature manifold. This gives us an idea of the stability and complexity of the decision boundaries that the model learned.

While deep learning-based ECG analysis has achieved state-of-the-art performance across various ECG classification tasks, comparatively less attention has been devoted to understanding how periodicity-aware temporal modeling influences representation learning quality, feature separability, class overlap reduction, decision boundary stability, and classifier confidence within complex multi-label ECG classification settings. Although existing TimesNet-based approaches demonstrate competitive performance in general time-series learning tasks, their application to periodicity-aware multi-label ECG representation learning remains comparatively less explored. In particular, the effect of increasing classification complexity on latent-space organization, feature discriminability, and prediction confidence within ECG-specific TimesNet frameworks has not been sufficiently investigated.

Given the nature of ECG signals, which contain quasi-periodic cardiac dynamics with temporal and morphology-related variations within and between periods, respectively, modeling the periodic structures within time-series is important for discriminative representation learning of ECG signals. In this study, we propose to integrate the idea of temporal periodicity with TimesNet-based multi-scale TimesBlocks to decompose the time-series into a number of intra- and inter-periodic components to capture the intra- and inter-period morphological variations within and between periods, respectively, for all multi-lead ECG recordings. In addition, to demonstrate how the incorporation of temporal periodicity affects the organization of features in the latent space, the study also examines decision boundaries and class separability of the learned representations using a number of techniques including UMAP, PCA, probability landscape, and a gradient-based feature importance mapping. The effects of periodicity-aware learning are further studied on a number of ECG classification tasks of varying complexities.

The main novelties and contributions of this work are summarized below.

1. In this paper, a periodicity-aware TimesNet-based framework is proposed for multi-label 12-lead ECG classification. With this architecture, rhythm-related temporal dependencies and morphology-related ECG characteristics are learned simultaneously.
2. For ECG analysis, FFT-guided temporal period extraction was incorporated to better capture periodic cardiac dynamics within multi-lead ECG recordings.
3. To capture both the short-term morphological variations and long-range temporal relationships in ECG time-series, we incorporated multi-scale TimesBlocks with different convolutional kernel sizes.
4. The proposed framework is evaluated using the widely adopted PTB-XL benchmark dataset with patient-level separation and standardized evaluation metrics to reduce information leakage and improve evaluation reliability.
5. In order to better understand the functionality of the proposed ECG classification method, a component-wise ablation analysis is provided. This analysis investigated the impact of several techniques, namely FFT-based period extraction, multi-scale temporal modeling, positional embeddings, and data augmentation.
6. To provide deeper insight into ECG representation learning, the proposed framework includes visualization and interpretability analyses using ROC curves, confusion matrices, UMAP and PCA latent-space visualization, probability landscapes, and gradient-based attribution mapping to investigate feature separability, prediction confidence, and physiologically relevant waveform regions influencing model predictions.
7. In addition to classification performance and representation learning analysis, the proposed framework was further evaluated through computational profiling, including inference latency, computational complexity, throughput, and memory utilization analysis to investigate practical deployment potential.

## 2. Proposed Method

**Figure 1:**
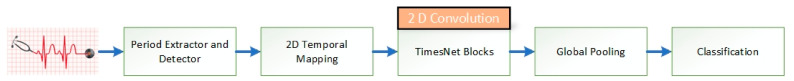
TimesNet architecture illustrating FFT-based period detection, 1D temporal mapping of time-series signals, feature extraction through TimesBlocks, global feature pooling, and final classification.

Figure 1 shows our model architecture; we developed and tested proposed systems that classify multiple ECG signals from the PTB-XL dataset. Although PTB-XL is a multi-label dataset, this work focuses on Three-Class (NORM, AFIB, PVC) and Five-Class (NORM, AFIB, MI, PVC, STTC) classification tasks instead of learning all the 15 possible diagnoses. The experiments are implemented in a one-vs-rest multi-label learning setting with sigmoid outputs where class-wise probability estimation is performed. In our experiments, we train and evaluate on a reduced set of diagnostic labels corresponding to the target classes that we are interested in. In principle, in the prediction of a recording, more than one target class can be activated, but for the purpose of confusion matrices and visualization, we consider the highest-class probability as the dominant predicted class for each recording and analyze its class-specific performance in detail. The target classes included normal ECG (NORM), AFIB, MI, PVC, and STTC.

Each preprocessed ECG sample was represented as $X_{i}\in R^{C\times T}$ to represent the preprocessed ECG signal for sample i, with C=12 leads and T=5000 time samples.

The multi-label target vector $y_{i}\in \{0,1{\}}^{K}$ is for K=5 classes represent the corresponding target vector. The PTB-XL database provides ECG recordings which exist in waveform database (WFDB) format. The accompanying dataset provided access to metadata which allowed us to use high-resolution waveform path when it was available; otherwise, they selected low-resolution waveform path. The original PTB-XL diagnostic statements were provided as SCP codes. This mapping process transformed the code into five diagnostic categories which clinicians could use for patient assessment, and it also decreased label sparsity. The original PTB-XL diagnostic annotations were provided as diagnostic SCP codes. These SCP codes were grouped into clinically relevant categories to reduce label sparsity. For example, all infarction-related codes IMI, AMI, ASMI, ALMI, INJAS, INJAL were assigned to the MI target class. All repolarization-related codes STTC, STD, STE were assigned to the STTC target class. The NORM, AFIB, PVC labels were directly mapped to the corresponding target classes.

$$y_{i}=[y_{i1},y_{i2},\ldots ,y_{i5}]\in \{0,1{\}}^{5},$$

The system uses the binary variable $y_{ik}=1$ to show which class k is present while $y_{ik}=0\;$ indicates the absence of that class. Only recordings containing at least one target diagnostic label were included in the analysis.

## 3. Signal Preprocessing

All ECG signals underwent standardization before the model training process. We began our work by loading the waveform data as multiple time-series channels. The deep network is trained on ECG signal time-series where the signal is first transformed into a matrix of temporal features that the network learns to extract and utilize for classification as shown in the below equation.

$$\tilde{X}\in R^{\tilde{T}\times \tilde{\;C}},$$

where $\tilde{T}$ is the original sequence length and $\tilde{C}$ is the original number of channels. Each signal was resampled to a common sampling frequency of 500 Hz using linear interpolation. This step ensured a uniform temporal resolution across all recordings. All ECG recordings were always resampled to 12 leads. Recordings with more than 12 channels were cut to 12 leads, recordings with less than 12 channels are extended by zero to 12 channels. All recordings have a fixed length of 5000 samples, which are center-cropped in case longer recordings are available and zero-padded in case shorter recordings are available.

## 4. Data Partitioning

The dataset was divided into three different subsets which included training and validation and test subsets. In order to avoid data leakage, the standard practice of time-series dataset partitioning at the patient level was followed. All available recordings of a single patient were thus assigned to either the training, validation or test subset. No data from the same patient was therefore available for evaluation on unseen data. This separation strategy is standard in time-series classification and follows the evaluation principles outlined in the original PTB-XL benchmark; it is also typically followed in ECG classification studies employing PTB-XL. For this reason, it enhances the ability to meaningfully assess generalization performance and to compare results with other approaches.

To tackle this challenging learning task, we employ a stratified partitioning strategy. We use the training set to tune the model’s parameters, and the validation set to tune model hyperparameters and to enforce early stopping to prevent overfitting. The test set is then used to evaluate the performance of the best performing model.

The PTB-XL dataset contains multi-label diagnostic annotations in SCP diagnostic code format. For this study we selected a subset of clinically relevant diagnostic categories to build the target classification tasks. Two different experimental settings have been investigated: a Three-Class setup (NORM, AFIB, PVC) and a Five-Class setup (NORM, AFIB, MI, PVC, STTC).

The original SCP diagnostic statements from the SCP file were mapped into the target diagnostic categories using clinically related grouping rules. Infract-related SCP codes, such as IMI, AMI, ASMI, ALMI, INJAS, and INJAL were grouped into the myocardial infarction (MI) category. Repolarization-related SCP codes such as STTC, STD_, and STE_ were grouped into the STTC category. Recording classifications of NORM, AFIB, and PVC annotations were used to map the SCP statements to the target classes.

As PTB-XL is a multi-label dataset, several target labels may be present in one recording at the same time. Following this, we encode target labels as multi-hot vectors and employ a one-vs-rest approach in multi-label learning. We exclude recordings that contain no target labels of the selected categories. In addition, recordings with missing waveform data or with leads other than mono, bipolar or tri-polarab were excluded in preprocessing/standardization step.

For visualization purposes, dominant-labeling was performed by selecting the class with the highest probability for each sample, and using this for the generation of confusion matrices and latent-space projections. However, all other aspects of training and evaluation were multi-label. For more information, refer to Table 1.

## 5. Data Augmentation

Implemented light augmentation for training data because they wanted to enhance generalization and decrease overfitting. The study employed three different techniques for augmentation. The first method involved temporal shifting, which required circular shifting the signal by a random number of samples drawn from a uniform range. The second method introduced additive Gaussian noise to the system. Third, lead dropout was used as channel-level regularization; with probability 0.25, one randomly selected lead was set to zero across all time points. The researchers applied mild augmentations because they wanted to maintain the physiological accuracy of ECG morphology while testing the model’s ability to handle small timing errors and noise and partial lead failure.

## 6. Model Architecture

**Figure 2:**
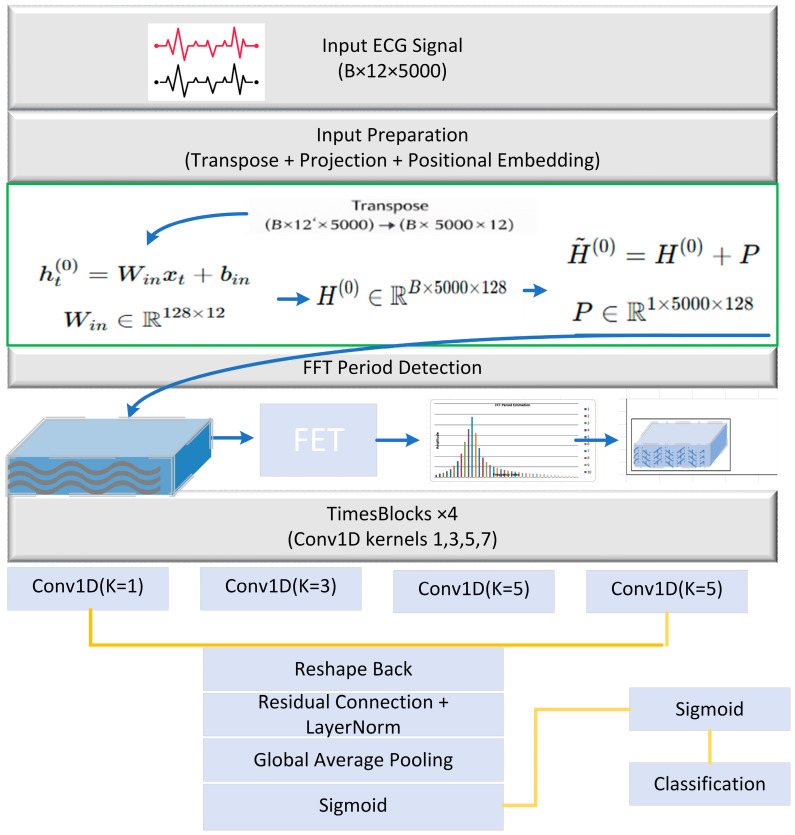
Illustration of the overall architecture of the proposed periodicity-aware TimesNet-based ECG classification framework.

The proposed model shown in Figure 2 adopts ECG-specific TimesNet-based framework architecture designed to capture both periodic and morphological patterns in multi-lead ECG signals. The model processes a batch of ECG recordings and transforms the raw signals into a latent representation that enables effective temporal feature learning through stacked TimesBlocks. The input ECG tensor is represented as follows: where B  is batch size, C=12  represents the number of ECG leads, and T=5000  denotes the temporal sequence length.

$$X\in R^{B\times C\times T}$$

The linear projection layer projects the raw ECG signal at each time step t into a low dimensional feature space.

$$h_{t}^{\left(0\right)}=W_{in}x_{t}+b_{in}$$

where $x_{t}\in R^{C}$: input vector at time step t, $W_{in}\in R^{D\;\times \;C}$: learnable projection matrix, $b_{in}$: bias term and D: latent embedding dimension (set to 128). The embedded sequence after projection transforms into the following representation, as summarized in Table 2.

$$H^{\left(0\right)}\in R^{B\times T\times D}$$

A learnable positional embedding, P, is added to the projected features to ensure that the temporal ordering information is preserved.

$${\tilde{H}}^{\left(0\right)}=H^{\left(0\right)}+P$$

where

$$P\in R^{1\times T\times D}$$

Positional encoding makes the model able to effectively learn from its surrounding information. ECG signals show quasi-periodic patterns which match the timing of heartbeats.

As quasi-periodic cardiac rhythms are characteristic for ECG signals, the model determines dominant temporal periodicities by calculating an FFT.

This is followed by a computation of the mean feature representation across latent channels:

$$v\in R^{B\times T}$$

The frequency spectrum is then obtained using

A=|FFT(v)|

The principal frequencies $f_{i}$ that come from the amplitude spectrum, and their corresponding temporal periods are computed.

$$p_{i}=\left\lfloor \frac{T}{f_{i}}\right\rfloor$$

These detected periods guide the temporal reshaping of the feature sequence within the TimesBlock.

The core architecture consists of four stacked TimesBlocks designed to learn representations across different periodic structures. The sequence is divided into period-aligned segments for each detected period $p_{i}$.

$$Z_{i}\in R^{B\times \frac{T}{p_{i}}\times p_{i}\times D}$$

This transition helps the model to capture various patterns within the individual heartbeats across the multiple cycles. The Inception-style parallel convolution module extracts features from TimesBlock content through its system of multiple Conv1D layers which operate at various kernel sizes k∈{1,3,5,7}. The kernels base their design on temporal dependencies which they extract from different time scales.

$$F_{1}=Conv_{k=1}(Z),{\;F}_{2}=Conv_{k=3}(Z),{\;F}_{3}=Conv_{k=5}(Z),{\;F}_{4}=Conv_{k=7}(Z).$$

The resulting feature maps are concatenated:

$$F=\mathrm{Concat}(F_{1},F_{2},F_{3},F_{4}).$$

Similarly, the output is reshaped back to be in the original temporal format and is then integrated back to the original tensor through a residual connection:

$$H^{\left(l\;+\;1\right)}=\mathrm{LayerNorm}(H^{\left(l\right)}+F).$$

The training process becomes more stable through this residual formulation which enables the system to maintain its existing learned patterns while it adds new temporal features. The final feature representation is obtained through global average pooling which operates on the entire temporal dimension after passing through the four TimesBlocks which are after passing through four stacked TimesBlocks.

$$H^{\left(l\right)}\in R^{B\times T\times D}$$

$$g\in R^{D}$$

The resulting pooled feature vector is then passed to a fully connected classification layer:

$$\hat{y}=\sigma (W_{c}g+b_{c})$$

The classification parameters are represented by $W_{c}$ and $b_{c}$, while the σ symbol stands for the sigmoid activation function which is used in multi-label prediction. The model outputs the probabilities for the target ECG classes, which include NORM, AFIB, MI, PVC, and STTC. Binary cross-entropy loss was optimized independently for each class to support one-vs-rest probability estimation within the multi-label framework.

## 7. Results and Discussion

In our framework, training was performed using a one-vs-rest multi-label setup; however, for class-wise visualization, confusion matrices and projections in latent space, a dominant-label assignment was done based on the highest predicted probability for ease of interpretation and comparison. ROC-AUC, precision, recall and F1-score are reported for each diagnostic class in the tasks using a one-vs-rest evaluation approach. Micro-averaged metrics that capture performance across all classes are also provided. The proposed TimesNet-based ECG classification framework was examined on the PTB-XL dataset through its five clinically significant diagnostic categories which included normal rhythm (NORM), atrial fibrillation (AFIB), myocardial infarction (MI), premature ventricular contraction (PVC) and ST-T abnormalities (STTC). The experimental pipeline produced a complete set of evaluation outputs which included training curves and ROC curves for training validation, testing datasets, per-class confusion matrices and detailed AUC statistics with confidence intervals [19].

**Figure 3:**
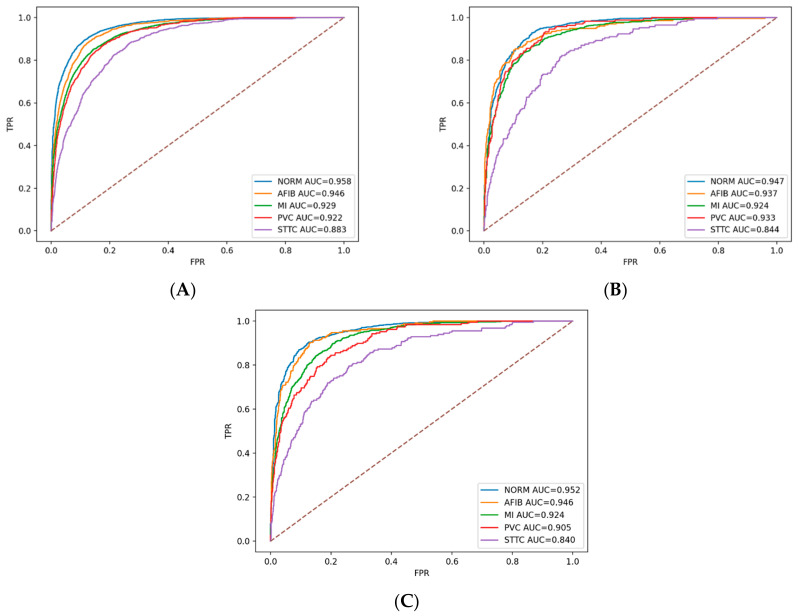
ROC curves for the Five-Class ECG classification using the TimesNet model on the PTB-XL dataset: (**A**) ROC curves obtained on the training dataset, demonstrating strong class separability and high discriminative capability across all five ECG classes; (**B**) ROC curves obtained on the validation dataset, showing the generalization performance of the proposed model during validation; (**C**) ROC curves obtained on the independent test dataset, illustrating the final classification performance and robustness of the model on unseen ECG recordings.

The model’s classification performance was evaluated through the Area Under the Receiver Operating Characteristic Curve (AUC-ROC) measurement together with precision, recall and F1-score assessment. The training, validation and testing datasets display their ROC curves in Figure 3A–C. The curves show how diagnostic categories achieve different levels of sensitivity and specificity through various decision thresholds. The ROC curves demonstrate strong discriminative capability for most classes. The normal rhythm and atrial fibrillation curves show steep pathways which lead to the upper left corner of the plot to demonstrate their high sensitivity and specificity. The myocardial infarction and premature ventricular contraction classes demonstrate strong ability to distinguish their positive and negative samples. The ST-T abnormalities show slightly lower separation abilities than the other classes because the condition requires clinical evaluation and ECG signals display subtle morphological changes.

The quantitative evaluation results are summarized in Table 3, which shows data from the test set. The mean AUC obtained across the five diagnostic classes on the independent test dataset was 0.913, which indicates strong overall classification performance.

Table 3 demonstrates that normal rhythm classification achieved the highest accuracy with an AUC score of 0.952. Atrial fibrillation received an AUC score of 0.946 as its second highest achievement. The results demonstrate that the model effectively differentiates between regular heartbeat patterns and irregular heartbeat patterns. The network succeeded in detecting myocardial infarction through its AUC result of 0.924, which proves that the system can recognize heart muscle damage patterns that occur during these medical events. The AUC score for premature ventricular contractions reached 0.905, which demonstrates the system’s capability to identify unusual patterns of ventricular depolarization that occur in ECG signals. The ST-T abnormalities produced the lowest AUC result of 0.840, which still demonstrates considerable predictive ability because of the intricate clinical nature of these patterns. The bootstrap confidence intervals confirm the statistical robustness of the results. The AUC for the normal rhythm class shows a confidence interval which extends from 0.944 to 0.961 because model performance shows minimal changes. The atrial fibrillation detection system exhibits a confidence interval between 0.932 and 0.959, which demonstrates its ability to maintain consistent predictive accuracy throughout various bootstrap tests.

The classification metrics found Table 4 provide additional information about model performance evaluation. The system achieved a micro-averaged precision of 0.700 while achieving a micro-averaged recall of 0.841 across all classes. The results produced a micro-averaged F1-score of 0.764, which shows an equal distribution between precision and recall. The model demonstrates good performance in finding pathological ECG patterns according to its high recall value, which may be useful in screening-oriented settings where sensitivity is important in situations that require accurate detection of all cases.

**Figure 4:**
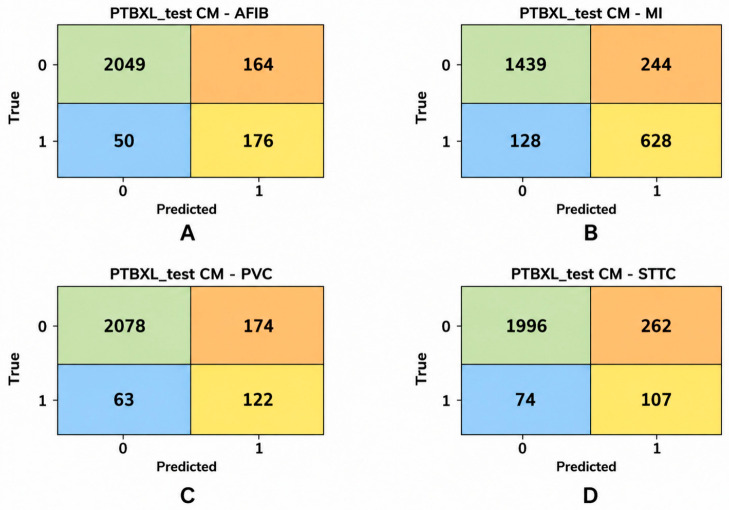
Confusion matrices for the Five-Class ECG classification on the PTB-XL test dataset: (**A**) AFIB; (**B**) MI; (**C**) PVC; (**D**) STTC.

The model achieved a 0.779 recall score for atrial fibrillation, which showed that the system successfully detected most AFIB cases. The confusion matrix from the study in Figure 4A shows that the model correctly identifies most AFIB recordings, but it occasionally misclassifies some cases as different arrhythmic conditions. The observation happens because atrial fibrillation shares specific waveform features with other irregular rhythm patterns. The F1-score for myocardial infarction classification reached 0.771, while the system achieved a precision rate of 0.720 and a recall rate of 0.831. The confusion matrix for MI (Figure 4B) shows that the majority of infarction cases are correctly detected, though some misclassifications occur with ST-T abnormalities due to overlapping morphological features.

The detection of premature ventricular contractions resulted in an F1-score of 0.505, which showed the challenge of identifying rare ventricular beats through continuous ECG monitoring. Confusion matrices were produced using the highest confidence score to classify each ECG recording to a particular class, even though the network has been trained on ECG recordings with multiple labels. The confusion matrix for PVC (Figure 4C) shows that some PVC events get incorrectly identified as other rhythm disorders because premature beats occur infrequently in the recorded signal. The most difficult testing situation occurred because ST-T abnormalities required classification. The model achieved an F1-score of 0.389 for this category, primarily due to lower precision values. The confusion matrix for this class (Figure 4D) shows that ST-T changes can be mistaken for myocardial infarction patterns because both conditions cause ventricular repolarization changes.

## 8. Three-Class

**Figure 5:**
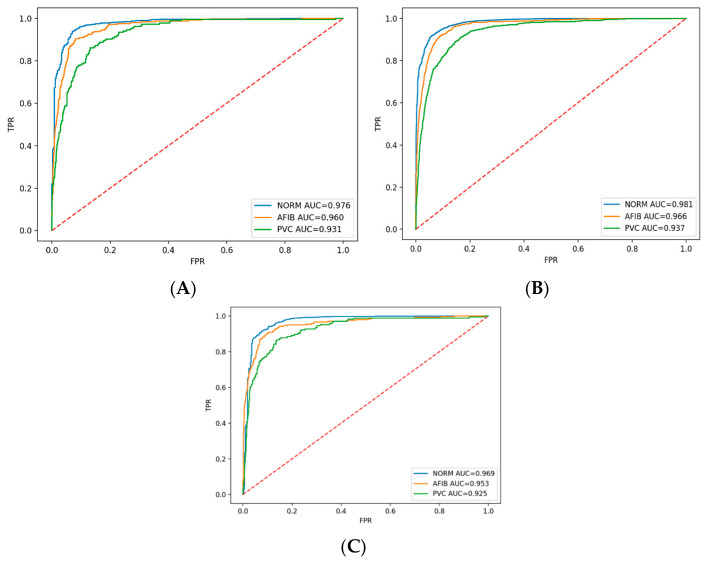
ROC curves showing the classification performance of the TimesNet model for NORM, AFIB, and PVC classes on the PTB-XL dataset: (**A**) Training dataset; (**B**) Validation dataset; (**C**) Test dataset.

The researchers assessed discriminative performance through receiver operating characteristic analysis across three dataset divisions. The training set ROC curves established through Figure 5A demonstrated exceptional diagnostic performance because all three diagnostic categories showed clear separability different from normal rhythm (NORM) atrial fibrillation (AFIB) and premature ventricular contraction (PVC) tracking. The validation set maintained strong separation which Figure 5B shows and the independent test set maintained this strength according to Figure 5C results. The three ROC figures show high agreement, which proves the model acquired temporal patterns that extended beyond the training dataset. This discovery holds particular significance for ECG analysis because temporal models in this domain typically overfit specific dataset signal patterns.

Table 5 presents the summary of quantitative performance metrics which were assessed on the test set. The model achieved a mean AUC of 0.9556, demonstrating strong discriminative performance across the three target diagnostic categories. The highest test AUC at the class level reached NORM with a score of 0.9763, whereas AFIB and PVC followed with scores of 0.9596 and 0.9308, respectively. The test set precision and recall results together with the F1-score measurements validate the effectiveness of the proposed framework. NORM achieved a precision rate of 0.9509 and a recall rate of 0.9801, which led to an F1-score of 0.9653 that demonstrates reliable identification of normal ECG recordings. The AFIB test results showed a precision of 0.7273 and a recall of 0.7635, which produced an F1-score of 0.7449 that showed strong classification abilities but faced difficulties when identifying atrial fibrillation patterns. The PVC model achieved a precision rate of 0.4348 and a recall rate of 0.8602, which produced an F1-score of 0.5776 because the model detected ventricular ectopic events at high rates but made excessive false positive errors for this category. The test set achieved a strong micro-averaged performance, which resulted in precision rates of 0.8317 and recall rates of 0.9394 together with an F1-score of 0.8823. The saved evaluation outputs provide direct support for these test metrics.

**Figure 6:**
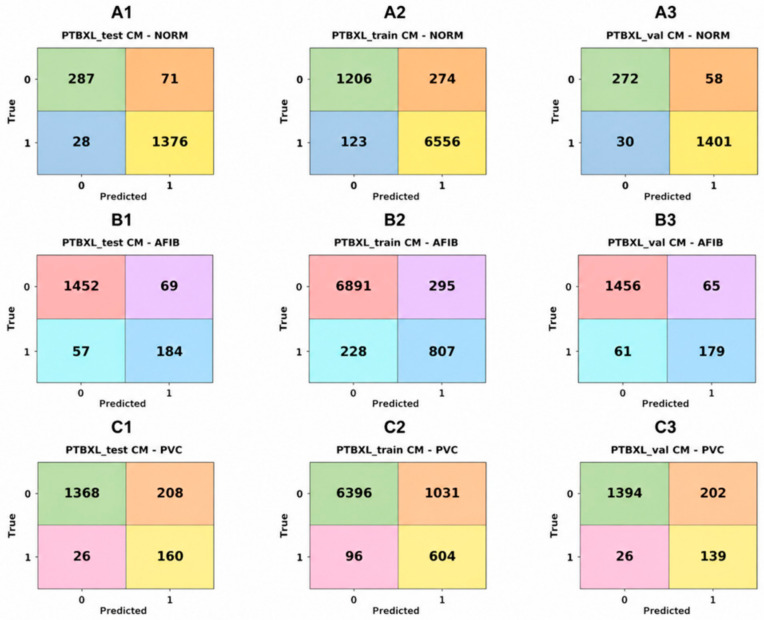
Confusion matrices for the Three-Class ECG classification on the PTB-XL dataset: (**A1**) NORM test dataset; (**A2**) NORM training dataset; (**A3**) NORM validation dataset; (**B1**) AFIB test dataset; (**B2**) AFIB training dataset; (**B3**) AFIB validation dataset; (**C1**) PVC test dataset; (**C2**) PVC training dataset; (**C3**) PVC validation dataset.

The NORM class displays excellent performance, which the confusion matrix analysis confirms. The main diagonal of NORM confusion matrices, which were tested across training validation and test sets, shows that most normal recordings were correctly identified according to the results in Figure 6A1–A3. The ongoing pattern stability through all data divisions proves that the model succeeded in developing strong normal ECG pattern recognition abilities. The class-wise analysis of the system explains its complete operational capacity. The model demonstrated exceptional accuracy in normal rhythm classification through its achievement of 0.896 precision, 0.914 recall, and 0.905 F1-score. The confusion matrix for this class, shown in Figure 6, indicates that the majority of normal ECG recordings are correctly assigned dominant-label predictions with very few false positives or false negatives.

The confusion matrices presented in Figure 6B1–B3 display balanced classification performance across the training and validation and test sets for AFIB detection. The confusion matrices demonstrate that true positive detection remained strong while off-diagonal errors occurred only to a limited extent. A frequency-aware temporal architecture like TimesNet can successfully capture the physiological evidence of atrial fibrillation, which creates rhythm irregularities and prevents organized atrial depolarization from occurring.

The PVC class proved to be the most difficult when tested through three different class categories. The confusion matrices for PVC show high sensitivity results, which produced lower precision results according to the evidence found in Figure 6C1–C3. The class-specific test results shown in Table 1 demonstrate that PVC reached the highest recall among all pathological classes but showed the lowest precision. The model successfully detected PVC-positive recordings yet it mistakenly identified many non-PVC recordings as positive results. The clinical understanding of this situation exists because PVC events can occur at irregular times and display different visual characteristics, which show only a small part of their total occurrence through a defined ECG segment. The model needs to increase its ability to detect ectopic ventricular activity because it functions under conditions where it must handle both false positive and true positive tests at the same time.

Although our framework achieves strong overall performance, certain classes receive poorer classification accuracy. Specifically, performance on STTC is worse than that of NORM and AFIB. Although less so, performance on PVC is also suboptimal. Several factors may account for the decrease in performance on these ECG subclasses. STTC patterns possess unique challenges, such as the variability in their morphology and the large overlap with other cardiac rhythm classes. Additionally, PVC patterns have great inter-patient variability, and even inpatient recordings may not follow a consistent temporal pattern. The class imbalance inherent to most rhythm classification datasets and the varying morphology of both normal and abnormal ECG signals further create challenges for representing these difficult ECG subclasses. Future work includes improving morphology-aware feature extraction techniques, leveraging class-balanced datasets, and enhancing temporal-context modeling for automatic ECG rhythm classification.

## 9. Ablation Study of Architecture Components

We performed an ablation study to highlight the functionalities of several components of our proposed framework. The full proposed model consisting of all the components has a mean AUC of 0.913 over all the 15 categories. We remove the FFT-based period extraction in TimesNet and the results in terms of mean AUC drops to 0.900, which highlights that frequency-aware temporal decomposition for learning of rhythm-related temporal dependencies are important for better classification performance. Also, removing multi-scale TimesBlocks results in a comparable drop in classification performance and this further highlights the importance of multi-scale temporal modeling for morphology-aware feature learning. Further, removing positional encoding and data augmentation results in a moderate decrease in classification performance, which is primarily due to the lack of temporal ordering information and insufficient representation learning and generalization of model. The ablation study thus highlights that frequency-aware temporal modeling integrated with multi-scale feature learning is crucial for achieving robust classification performance for automated multi-label ECG classification (Table 6).

## 10. Why the Three-Class Configuration Produced Better Results than the Five-Class Setup: Evidence from Visualization and Model Interpretability

This improved performance on Three-Classes was further substantiated by ECG visualization and gradient-based gradient maps. The grouped visualizations suggest that reducing the number of classes allowed the network to learn more separable representations and consistent decision patterns for different classes than it was able to in the more challenging Five-Class setting.

**Figure 7:**
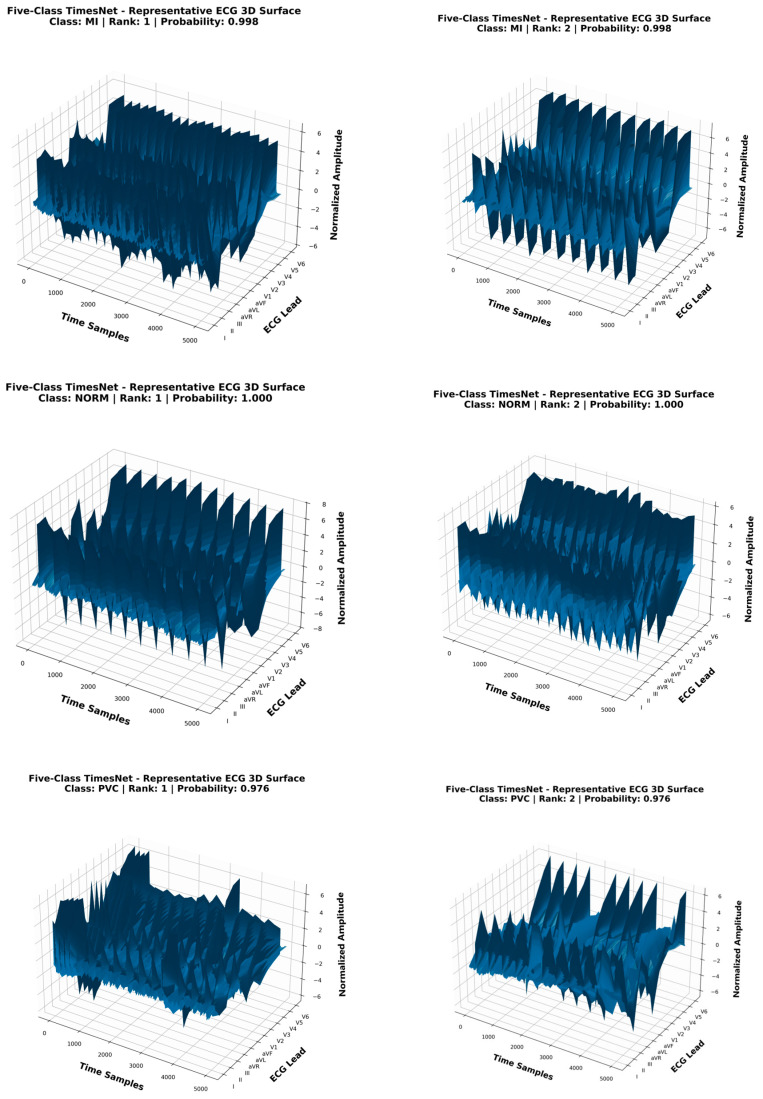

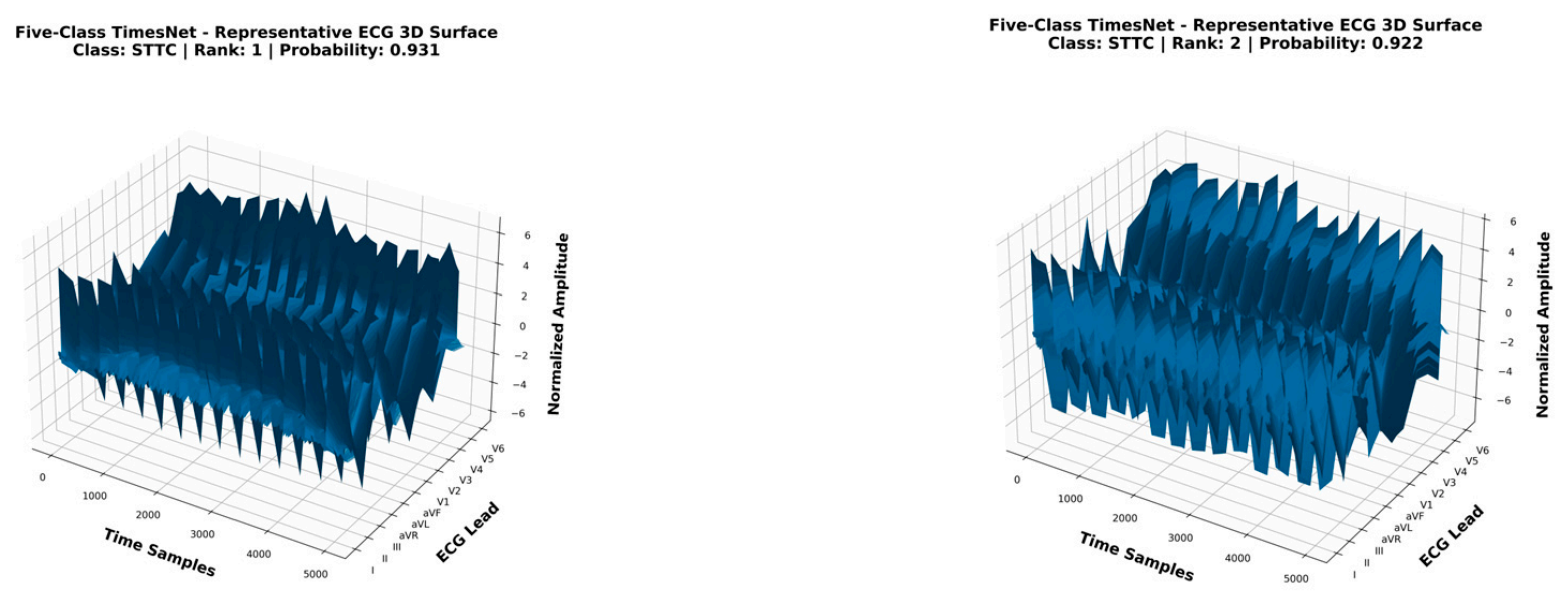
Representative 3D ECG surface visualizations for the Five-Class configuration, showing signal amplitude across time–lead dimensions for NORM, AFIB, MI, STTC, and PVC classes, highlighting morphological similarities between MI and STTC patterns.

Figure 7 displays representative ECG recordings, which demonstrate five different classes of the study. The signal examination demonstrates that multiple pathological conditions exhibit identical waveform patterns. The myocardial infarction recordings show waveform patterns which match the ST-T aberration recording. Both conditions display similar deviations in the ST segment and T-wave morphology. The ECG patterns of these repolarization abnormalities show multiple similarities because these abnormalities stem from similar physiological processes. The classification task encounters challenges because the overlapping patterns between two classes create uncertainty for the model to establish distinct feature boundaries that separate MI from STTC classes.

Figure 7 displays the 3D ECG surface visualizations which represent the Five-Class configuration through the display of samples NORM, AFIB, MI, STTC and PVC. The 3D surface plots display ECG signal amplitude measurements which extend across both time and lead dimensions to create a space-based view of waveform changes and waveform shape changes. The overlapping morphological patterns between the classes make it harder to separate them, which results in higher chances of misclassification during Five-Class testing, as shown by the surface similarity patterns in Figure 7.

**Figure 8:**
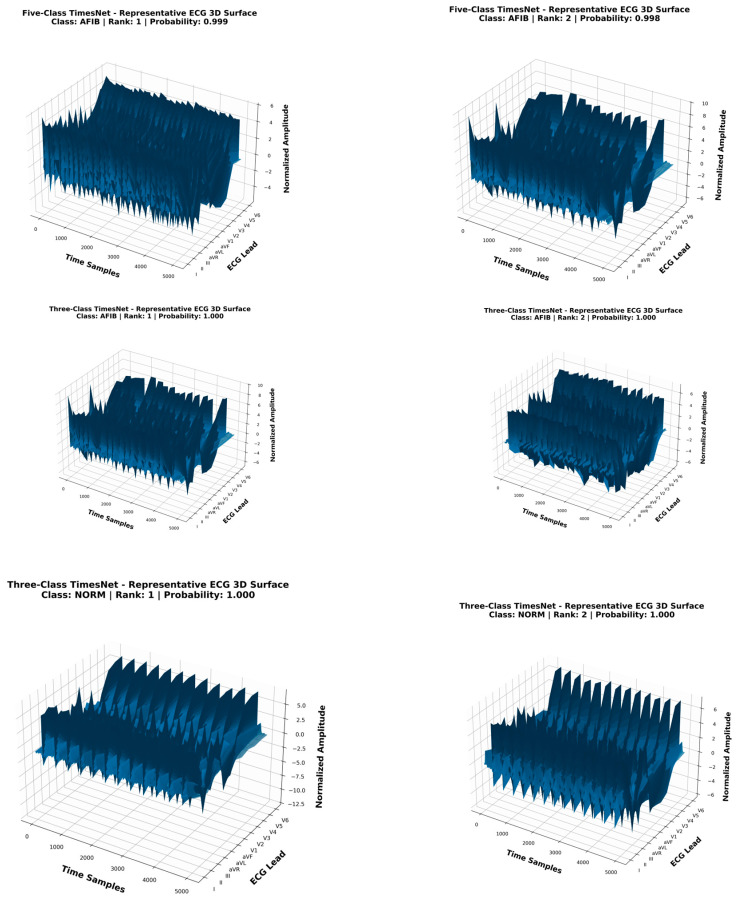

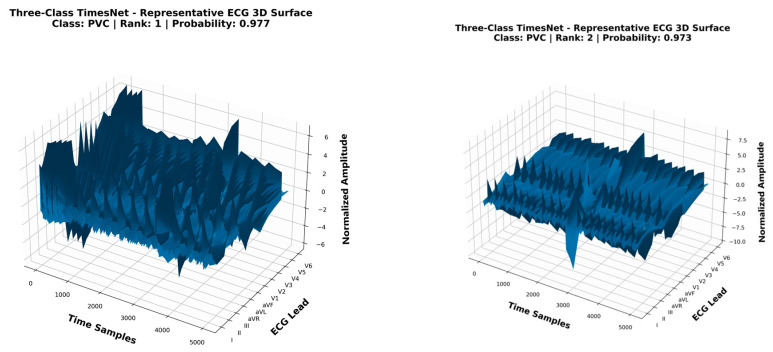
Representative 3D ECG surface visualizations for the Three-Class configuration (NORM, AFIB, PVC), illustrating distinct temporal–morphological patterns across the time–lead plane and demonstrating clearer structural separability between rhythm-based cardiac conditions.

ECG surface displays for the Three-Class data (NORM, AFIB and PVC) are shown in Figure 8. In contrast to the Five-Class scenario, the waveform patterns and temporal dynamics are more distinct and visible for each class, visually supporting the enhanced feature separation for classification observed in the previous section. The NORM recordings display stable cardiac cycles which show regular R–R intervals and maintain consistent waveform patterns throughout the time–lead plane. AFIB signals show distinct rhythm disturbances because their atrial rhythms develop through uncoordinated atrial electrical activity. The PVC recordings show premature ventricular depolarization events which create QRS complexes that extend beyond their normal width and result in brief periods of time where the heart rhythm pattern becomes disordered. The 3D time–lead visualization shows that these rhythm disturbances produce specific surface patterns which display strong contrast to standard cardiac functions. The TimesNet-based architecture is designed to model periodic and temporal structures for modeling periodic structures together with temporal patterns in time-series data, which enables it to capture rhythm-related abnormalities. The Three-Class system enables better feature space separation, which results in more stable decision boundaries and enhanced classification results.

## 11. Gradient-Based Model Interpretability

The gradient-based interpretability maps show how the model operates, which gives researchers more information about its functioning. The visualizations display which parts of the ECG signal have the strongest effect on the model’s prediction.

**Figure 9:**
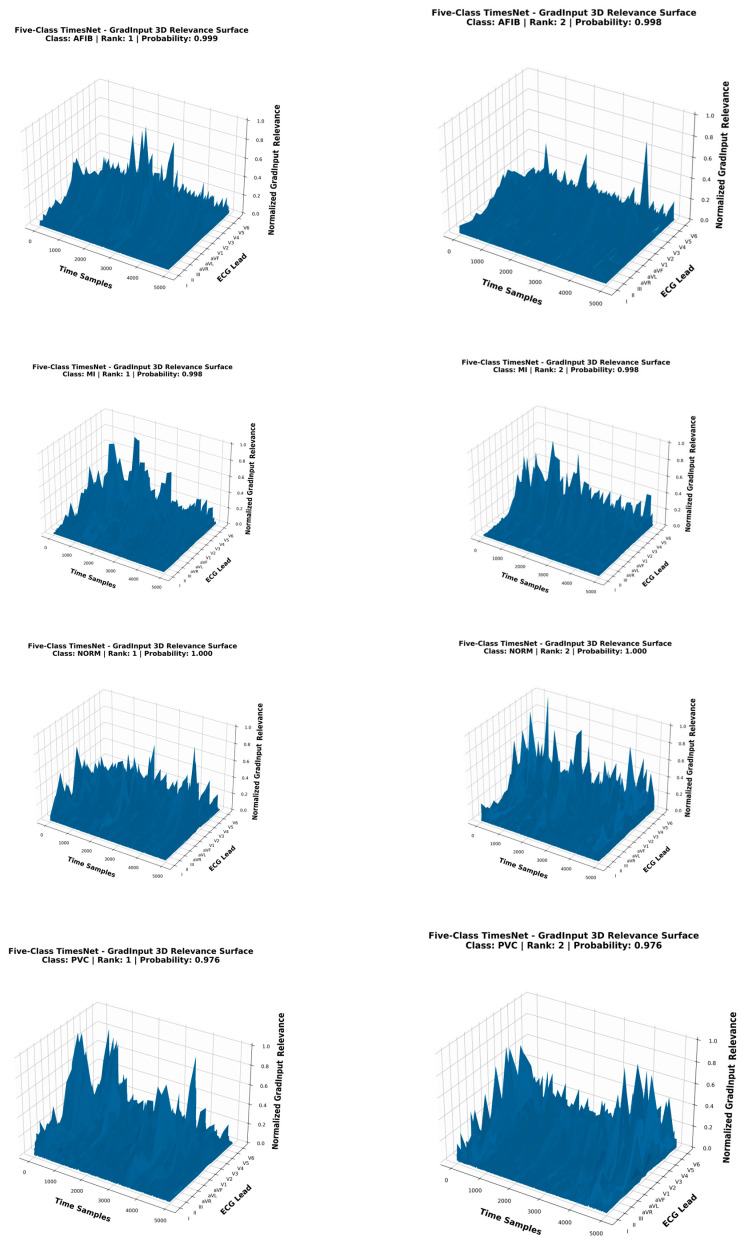

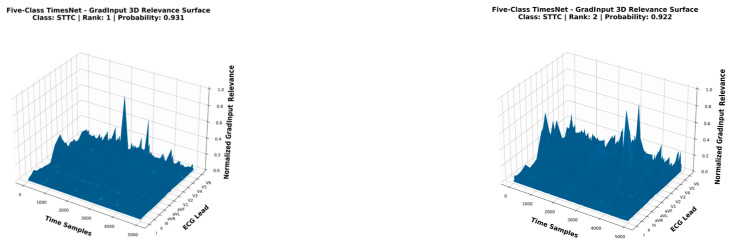
GradInput attribution maps for the Five-Class configuration, illustrating the regions of the ECG signal that most strongly influence model predictions for NORM, AFIB, MI, STTC, and PVC classes. Similar attention patterns between MI and STTC indicate overlapping morphological features.

The Five-Class model GradInput attribution maps appear in Figure 9, which displays NORM, AFIB, MI, STTC and PVC. The model demonstrates a pattern of repeated attention to the same waveform areas during its examination of myocardial infarction and ST-T abnormality signals. The regions which receive emphasis in MI GradInput show a strong focus on the ST segment and T-wave areas of the ECG signal. The same attention pattern appears STTC GradInput. The model uses identical signal segments for its MI and STTC classification, which leads to challenges in distinguishing learned feature representations. The two categories exhibit classification errors in the Five-Class experiment because of their overlapping features. Figure 9 results show that multiple disease categories display identical surface pattern structures which they use to identify different diseases. The myocardial infarction surface MI shows surface patterns that contain both amplitude changes and structural defects which match the patterns observed in ST-T abnormality surfaces STTC. The time–lead plane shows both surface sets with matching elevation and depression patterns that demonstrate identical repolarization disturbances present in the underlying ECG signals. The two diagnostic categories use waveform patterns that create overlapping regions because both categories share similar electrophysiological attributes. The deep learning model faces challenges when trying to create distinct boundaries for classifying MI and STTC because of this problem. The learned representations show increased feature overlap because of structural similarity, which makes the classifier depend on tiny differences that are hard to identify.

**Figure 10:**
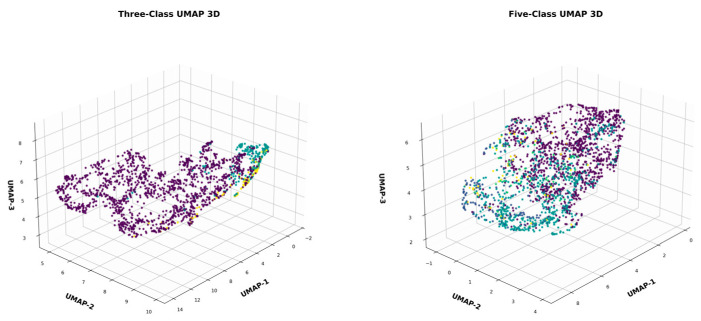
3D UMAP visualization of the learned latent feature space. The Three-Class model shows clearer cluster separation, whereas the Five-Class model exhibits greater overlap among pathological classes.

The GradInput attribution maps which show Three-Class model results are displayed in Figure 10, which contains NOR, AFIB and PVC. In these visualizations, the model shows more concentrated attention patterns which focus on specific classes. The circuit diagram shows AFIB GradInput1, which identifies irregular rhythm segments that result from R–R interval changes which mark the main electrophysiological feature of atrial fibrillation. The PVC GradInput1 signal highlights the specific ventricular beats which enable identification of PVC recordings that differ from standard rhythm patterns. The model achieves better performance through its ability to learn distinct waveform characteristics which each class uses as its unique identifying features.

**Figure 11:**
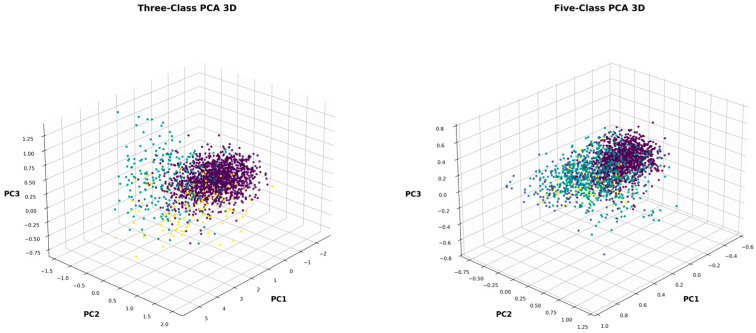
3D PCA projection of learned feature representations. The Three-Class configuration exhibits more compact clusters, whereas the Five-Class configuration shows increased overlap among classes.

Figure 10 and Figure 11 demonstrate their ability to provide qualitative proof which explains why Three-Class systems show better performance results. The Five-Class experiment showed that the model displayed similar attention patterns to MI and STTC diagnostic categories because both categories showed almost identical waveform characteristics. The similarity between two elements causes confusion about which features to show because it creates a challenge in defining consistent decision-making thresholds. The Three-Class system identifies rhythm-based abnormalities which exhibit unique temporal patterns throughout their detection process. The representative ECG signals and gradient attribution maps show that the model learns clearer and more physiologically meaningful features for NORM, AFIB, and PVC signals. We observe improved performance in the Three-Class problem possibly due to reduced class overlap and improved feature separability in the latent space as opposed to the highly complex Five-Class problem. The TimesNet-based framework leverages this reduced classification complexity to learn class-specific temporal dynamics that are more consistent and achieve higher AUC scores and F1-scores, with overall classification performance more consistent across the evaluated metrics. 

## 12. Visualization Analysis and Insights

The TimesNet model’s internal feature representations were studied through different dimensionality reduction visualizations which used UMAP and PCA. The network extracted high-dimensional latent features which the visualizations projected into three-dimensional space for qualitative assessment of class separability. The Three-Class UMAP representation shows three diagnostic categories (NORM, AFIB, and PVC) through distinct clusters that emerge from the projected samples. The model has learned to distinguish between normal rhythms and arrhythmic patterns because the clusters show compact structures with limited overlap. The NORM cluster creates a dense area which AFIB and PVC samples occupy; they occupy separate areas throughout the latent space. The model succeeds in capturing rhythm irregularities and morphological deviations that are linked to these conditions according to the separation between the two groups. The Five-Class UMAP representation demonstrates more cluster overlap than the previous method. Pathological samples show intersection between their two patterns of distribution while normal samples stay mostly together. The myocardial infarction (MI) and ST-T abnormalities (STTC) clusters show high levels of connection between each other. The two conditions share similar feature characteristics which create challenges for the classifier to define distinct decision boundaries. The latent space becomes less separable because five diagnostic categories require more effort to differentiate between them. The observations demonstrate through visual evidence that the Three-Class configuration creates a more organized and distinct feature space, which leads to better classification results (refer to Figure 10).

The PCA visualizations serve as evidence that the network has acquired its hidden representation structure. The PCA projection for the Three-Class configuration shows separate sample groups which contain only minimal overlap between different classes. The PCA dimensionality reduction method shows less capacity to represent data than UMAP, yet it still maintains visible cluster separation. The learned features show strong ability to separate different classes because they maintain their distinctiveness through linear projection. The PCA projection shows overlapping distributions between four pathological categories in the Five-Class configuration. The MI and STTC clusters show partial merging because their extracted features share common morphological characteristics. This observation matches established clinical relationships between ST segment and T-wave abnormalities and myocardial injury, which show similar ECG waveform patterns. (Refer to Figure 11).

**Figure 12:**
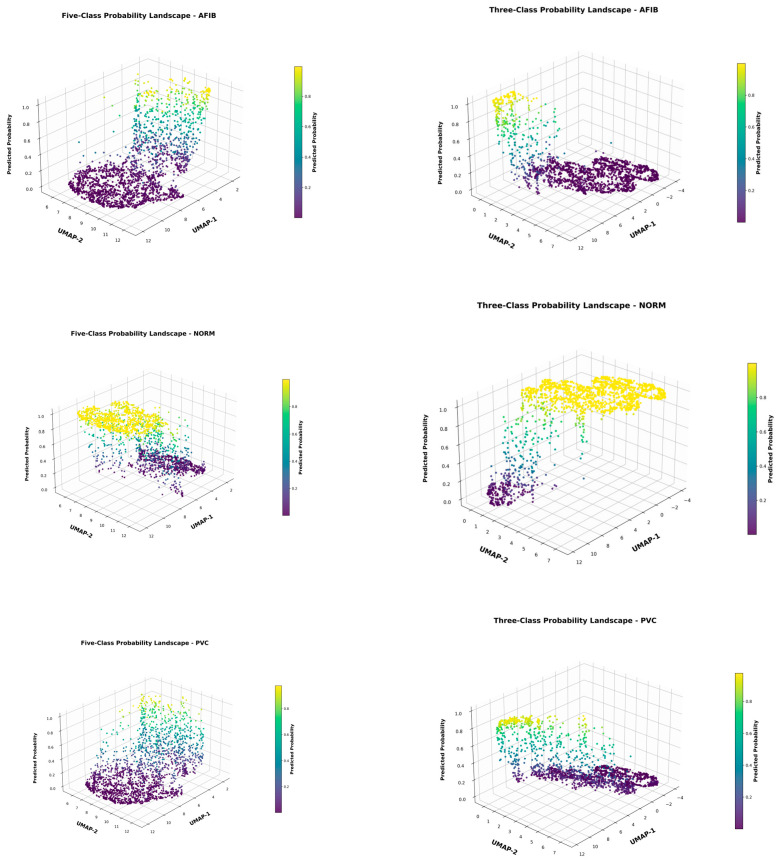
Probability landscape visualization of ECG classification in the learned feature space, where elevation represents predicted class probability for AFIB, NORM, and PVC samples.

The visualizations of probability landscapes show how the classifier interprets the feature space that it has learned. The Three-Class model uses probability surfaces which create distinct areas that each class controls in the feature space. The class boundaries show a smooth stable pattern which allows the classifier to make accurate predictions for all classes. The Five-Class probability landscapes show irregular boundary patterns which create disjoint probability areas throughout the landscape. The decision surfaces for certain classes overlap substantially, particularly between MI and STTC categories. The classifier needs to determine multiple intricate decision boundaries because it must differentiate between these interconnected pathological patterns (refer to Figure 12).

## 13. Computational Complexity and Scalability Analysis

The computational complexity and scalability characteristics of the proposed periodicity-aware TimesNet framework are summarized in Table 7. The proposed periodicity-aware TimesNet framework contains approximately 1.957 million trainable parameters. Computational profiling demonstrated that the framework required approximately 13.14 GFLOPs per ECG recording during inference. For a 12-lead ECG signal consisting of 5000 samples, the framework achieved an average inference latency of 5.230 ms per recording using CUDA GPU hardware, corresponding to a throughput of approximately 191.21 ECG recordings per second. The median inference latency was 5.229 ms with a low standard deviation of 0.064 ms, indicating stable inference performance. Peak allocated GPU memory and reserved GPU memory during inference were approximately 55.17 MB and 76.00 MB, respectively.

These findings indicate practical potential for scalable offline and cloud-assisted ECG analysis applications. The relatively low inference latency together with moderate memory utilization suggests that the framework can efficiently process large-scale ECG datasets and may support future deployment in real-time clinical decision-support systems and remote cardiac monitoring platforms operating in realistic healthcare environments.

Compared with many recently reported transformer-based and recurrent ECG classification frameworks, which often require substantially higher computational complexity, memory utilization, and inference latency, the proposed periodicity-aware framework maintains relatively efficient computational performance while preserving competitive classification accuracy and interpretable temporal feature representations. Specifically, the framework achieves approximately 13.14 GFLOPs computational complexity, moderate GPU memory utilization of 55.17 MB, and fast inference latency of 5.230 ms per ECG recording, making it suitable for scalable automated ECG analysis applications.

In addition, the interpretability of the proposed framework was investigated using UMAP and PCA latent-space visualization, probability landscape analysis, confusion matrix analysis, and gradient-based attribution mapping. These analyses provide insight into feature separability, class overlap reduction, decision boundary stability, and physiologically relevant waveform regions influencing model predictions, thereby improving the transparency and explainability of the ECG classification process.

Although the proposed framework demonstrated favorable computational efficiency on GPU hardware, additional optimization and validation on resource-constrained edge devices and wearable platforms remain necessary before large-scale real-time deployment in low-power clinical environments. Furthermore, the proposed framework is intended to support automated ECG interpretation and assist clinicians during diagnostic assessment rather than replace expert cardiologist evaluation.

## 14. Literature Survey

Table 8 shows a state-of-the-art methodology from the referenced studies, emphasizing their key approaches and performance metrics. In [22], the authors proposed a transformer-based deep learning model called MTDL-Net for the classification of ECG heartbeats. By utilizing the advantages of masked attention embedding in deep learning for extracting the morphological characteristics of ECG signals and a new temporal feature enhancement mechanism for analyzing the heartbeat dynamics, the proposed MTDL-Net effectively learns the ECG spatiotemporal features and therefore achieves a high classification performance. The experimental results demonstrate the efficiency of the proposed approach with the deep learning models yielding an average accuracy of 95.6%, a maximum specificity of 98.7% and a maximum recall of 96.4%. In [23], an inter-patient arrhythmia classification approach based on an ensemble machine learning approach that uses two ECG leads to classify five distinct morphological arrhythmias, namely LBBB, RBBB, PVC, PAC and Normal. The proposed approach includes ECG signal processing, feature extraction, feature selection and hyperparameter tuning. The proposed approach achieved an average accuracy of 87%, a sensitivity of 87.4%, a precision of 88.4% and an F1-score of 87%, which is a more competitive performance than the existing literature. Authors propose Morphology–Rhythm Contrast (MRC) [24], a contrastive learning framework for multi-lead ECG representation learning based on morphology and rhythm-based augmentations utilizing a triple-branch network. Linear probing achieves strong performance across the PTB-XL, CPSC and Chapman datasets with an average AUROC of 0.9889 and AUPRC of 0.9694, outperforming random initialization and a range of supervised baselines. The authors [25] propose a novel transformer-based model named Multi-Scale Grid Transformer (MSGformer) for ECG arrhythmia classification. The proposed model not only combines the strengths of multi-lead spatial feature fusion in ECG signal processing but also the multi-scale grid attention mechanism, to extract and learn the temporal and morphological patterns of ECG signals. The proposed model obtains satisfactory performance with an F1-score of about 0.86 on CPSC-2018 dataset, and 99.28% accuracy, 97.13% sensitivity, and 97.87% positive predictive value (PPV) on MIT-BIH dataset, which are all more competitive performances than those obtained by other state-of-the-art approaches. In [26], they present a hybrid deep learning approach for ECG classification that leverages the merits of 1D-CNN with squeeze-and-excitation attention for adaptive multi-scale feature learning. The proposed model incorporates the loss function of focal loss, L2 regularization and ensemble mixed-precision training. Experimental results achieved 99.48% accuracy on MIT-BIH, 99.83% on PTB and 99.64% on the combined dataset with F1-score reaching up to 1.00. In [27], the authors investigated a novel approach of ECG classification using deep learning, specifically by employing Restricted Boltzmann Machines (RBMs) and Deep Belief Networks (DBNs). Authors used the MIT-BIH database of recordings for the investigation, which allowed us to train our network to classify ventricular and supraventricular ectopic beats while reaching classification accuracy of 93.63% and 95.57%, respectively, at a very low sampling frequency of 114 Hz. The deep learning approach allowed for a quick, easy and accurate classification of the ECG signals by using simple features and reducing the data complexity, which makes this a very efficient and scalable approach for ECG classification. In [28], the authors proposed Y-Net, a deep learning-based approach for the robust ECG segmentation with the capability of learning single-lead and multi-lead ECG signals. Y-Net is developed with a dual-branch structure and a two-stage training scheme to deal with different rhythm types. In the experiments, the authors evaluated the performance of Y-Net on the LUDB and RDB datasets and the results showed the highest F1-scores as 99.60% and 99.03% within intra-dataset and inter-dataset evaluation respectively. Meanwhile, the AF detection based on morphological features extracted from the segmentation results of Y-Net showed the highest AUC as 0.983, indicating that the proposed method is not only effective but also interpretable and clinically practical. In [29], the authors proposed DeepMI, a deep learning based end-to-end framework to detect myocardial infarction (MI) and occurrence-time (Acute, Recent, Old) from 12-lead ECG signals. The DeepMI network adopted multi-level fusion (data, feature, decision), coupled with the advantages of pre-trained networks and recurrent layers, to extract the temporal information from ECG signals. DeepMI was validated in 17,381 patients and achieved the AUROCs of 96.7% (Normal), 82.9% (Acute), 68.6% (Recent), and 73.8% (Old) for multi-lead ECG-based analysis and the temporal classification of MI. In [30], the authors proposed a new machine learning approach designed to diagnose Left Ventricular Hypertrophy (LVH) from multi-lead ECG signals acquired from patients. The clinical features associated with ECG signals were extracted using the continuous wavelet transform, and peak detection of R and S waves in the ECG signal. Different classifiers such as kNN, decision trees, Naive Bayes, support vector machines, and neural networks have been used in this study. Experimental results revealed that neural networks achieved the highest accuracy, which was 97.8% on average. The authors thus propose an efficient cost-effective method to diagnose cardiac problems [30]. In [31], a novel deep learning method that employs a CNN model to extract mixed-scale hierarchical features from ECG signals and a Lead Encoder Attention (LEA) mechanism to fully utilize the morphological and temporal information of ECG is developed to classify multi-lead arrhythmia. Experimental results on MIT-BIH and CCDD datasets confirmed the effectiveness and robustness of the proposed method with the highest accuracy of 99.5% achieved by the proposed method on MIT-BIH and an average accuracy of 88.5% with good cross-dataset generalization. Authors [32] introduced a two-stage deep CNN model for efficient detection of hypertension and corresponding risk assessment of hypertensive patients from the multi-lead ECG signals. In the proposed work, the first stage is dedicated to detecting hypertension from the given ECG signal, while the second stage is responsible for risk assessment of detected hypertensive patients and classification of low- and high-risk patients. Extensive experiments with benchmark public datasets yielded remarkable performances, of 99.68% and 90.98% accuracy in detection and risk assessment stages, respectively. The excellent performances make the proposed approach eligible for clinical practice that can be efficiently moved to a cloud infrastructure. In [33], the authors show that deep learning models can achieve high accuracy in ECG analysis. However, those models usually need a large amount of labeled data to train, which is expensive and hard to obtain. In this work, they proposed a self-supervised generative pretraining approach called ECG-MAE. They use the model to learn the spatiotemporal representation of ECG by training it to restore masked regions of 12-lead ECG over time and leads. When fine-tuning the model for multi-label classification tasks, it significantly outperforms previous models and achieves a higher macro-AUC (0.9474) with higher label efficiency, better performance on the test sets and more capability to diagnose rare cardiac conditions. In [34], they designed and evaluated a one-dimensional deep densely connected neural network (DDNN) to classify atrial fibrillation (AF) using 12-lead ECG signals. Experiments on 16,557 recordings yield an accuracy of 99.35%, a sensitivity of 99.19% and a specificity of 99.44%, showing robust and high-performance classification. Their model holds great promise for clinical diagnostics and for use in wearable screening devices for AF.

Table 8 compares state-of-the-art approaches to ECG classification and situates the proposed TimesNet-based-based framework in the spectrum of deep learning and machine learning techniques. The methods developed in prior ECG classification works all employ deep learning and machine learning techniques, including transformer networks (e.g., MSGformer, Table 8), CNNs (e.g., hierarchical architecture in Table 8), RNNs (e.g., Table 7), and self-supervised learning (e.g., ECG-MAE, Table 8). Many of them have reached state-of-the-art classification performance on various datasets, in many cases even for challenging multi-class classification tasks. For example, very high classification accuracies have recently been reported for binary classification tasks as well as for classification tasks with limited numbers of labels (few-shot learning) using multi-scale temporal and morphological feature extraction. For learning effective ECG representations, self-supervised pretraining on large-scale datasets has also been shown to be very effective for efficient classification with limited labeled data on the challenging PTB-XL dataset.

Although significant progress has been achieved for various ECG classification tasks, there is still a major barrier for direct comparison between different studies, resulting from huge differences in several aspects, such as data construction, lead settings, class definitions, data preprocessing and augmentation strategies, evaluation metrics, and classification settings. Some studies focused on binary arrhythmia detection or atrial fibrillation screening, while others tackled specific clinical scenarios such as distinguishing healthy subjects from those with serious cardiac diseases. In this work, we evaluate the proposed periodicity-aware framework using a multi-label PTB-XL setting with patient-level data separation and standardized evaluation procedures. The primary focus of the study is on periodicity-aware ECG representation learning, interpretability analysis, and computational feasibility rather than claiming novelty in PTB-XL classification itself. Unlike existing methods, our method incorporates periodicity-aware temporal modeling to leverage time-sensitive features from ECG signals within the TimesNet framework. Our approach, built upon FFT-based period extraction and multi-scale TimesBlocks, effectively captures both rhythm-related temporal relationships and morphology-related waveform features within multi-lead ECG recordings. Through an ablation study, we demonstrate the effectiveness of frequency-aware temporal decomposition and multi-scale feature learning for ECG classification. While high classification accuracies have been reported in recent works under constrained binary or limited multi-class settings, our approach achieves competitive performance on a more challenging multi-label classification task on ECG recordings that better reflect clinical scenarios. Beyond classification performance, our framework incorporate periodicity-aware temporal structure learning into deep models and facilitates improved interpretability analysis for ECG-based health monitoring via both latent-space and gradient-based analysis methods. While deep ECG classification methods have achieved state-of-the-art results on several tasks in electrocardiogram classification, current methods have several limitations. Although substantial progress has been achieved in ECG classification using PTB-XL and related datasets, direct comparison between studies remains challenging because of differences in lead configurations, class definitions, preprocessing strategies, evaluation metrics, and experimental protocols. Furthermore, comparatively less attention has been devoted to understanding how periodicity-aware temporal modeling influences representation learning quality, feature separability, class overlap reduction, interpretability, and computational scalability within ECG-specific multi-label classification settings.

## 15. Conclusions

In this paper, we have developed a framework for multi-label ECG classification using deep neural networks. In our implementation, we used a one-vs-rest multi-label learning approach where sigmoid function-based probabilities for each class were computed separately. For visualization and convenience of confusion matrix analysis, dominant-label was assigned by selecting the class with the maximum probability. It capitalizes on the periodic nature of ECG signals to facilitate the extraction of temporal features from the signals. With the aid of the FET for the period detection and multi-scale convolutional features in TimesBlocks, it comprehensively extracts intra-period morphological features and inter-period rhythm features from multi-lead ECG signals to achieve the desired performance. Experimental results are provided over the PTB-XL dataset and the obtained classification performance was competitive, achieving a mean AUC of 0.913 and 0.956 for the Five-Class and Three-Class settings respectively. Furthermore, it is demonstrated that class complexity reduction leads to higher feature separability, stability of classification decision regions and finally to higher model robustness. Finally, visualizing the clusters of different classes over UMAP and PCA representations shows that three class setting leads to more separable and thus more pure clusters, while gradient-based explanations demonstrate that the model focuses on the physiologically meaningful representations of heart beats especially for the rhythm-related pathologies like AFIB and PVC.

While the model achieved a competitive performance, the authors also identified some limitations. These were mainly to do with classifying more morphologically similar conditions, such as myocardial infarction and ST-T abnormalities, within the Five-Class setting. The authors argue that there are data-driven restrictions when it comes to identifying the subtle changes in repolarization associated with these cardiac conditions.

Future work will focus on several key directions. First, integrating morphology-aware attention mechanisms or hybrid architectures combining TimesNet with clinical feature extraction may improve discrimination between overlapping classes such as MI and STTC. Second, incorporating multi-modal data (e.g., clinical metadata, patient history, or imaging) could enhance diagnostic robustness.

## Figures and Tables

**Table 1:** Summary of the PTB-XL dataset characteristics and the customized Five-Class ECG classification setting used in this study.

Category	Description
Dataset Name	PTB-XL Electrocardiography Dataset
Source	PhysioNet/PTB-XL
Total ECG Recordings	21,837 clinical ECG recordings
Number of Patients	18,885 patients
ECG Type	12-lead diagnostic ECG
Recording Duration	10 s ECG recordings
Sampling Frequency	500 Hz (100 Hz down sampled version also available)
Annotation Type	Expert cardiologist annotations based on SCP-ECG statements
Classification Setting Used	Customized Five-Class setting
Selected Classes	NORM, AFIB, MI, PVC, STTC
Label Nature	Multi-label diagnostic and arrhythmia annotations
Preprocessing	Signal normalization and label-specific grouping applied before training
Dataset Characteristics	Large-scale heterogeneous ECG dataset

**Table 2:** Mathematical symbols, tensor dimensions, and indexing notation used in the proposed ECG-specific TimesNet architecture.

Symbol	Meaning
(B)	Batch size
(C)	Number of ECG leads/channels
(T)	Number of temporal samples (time steps)
(D)	Latent embedding dimension
(L)	Number of stacked TimesBlocks
(M)	Number of target ECG classes
(r)	Index of detected dominant temporal period/frequency
(q)	Convolution kernel-size index
(m)	Target class index
(p_r)	Temporal period corresponding to frequency (f_r)
PTBXL(_{train})	PTB-XL training dataset split
PTBXL(_{val})	PTB-XL validation dataset split
PTBXL(_{test})	PTB-XL independent test dataset split
pos_support	Number of positive samples belonging to a target class
AUC(_{CI_low})	Lower bound of the confidence interval for AUC
AUC(_{CI_high})	Upper bound of the confidence interval for AUC

**Table 3:** AUC performance with 95% confidence intervals for the TimesNet model on the PTB-XL test set across five ECG classes.

Split	Class	AUC	AUC_CI_Low	AUC_CI_High	Pos_Support
PTBXL	NORM	0.952463	0.944135	0.960705	1412
PTBXL	AFIB	0.946267	0.932102	0.958759	226
PTBXL	MI	0.923796	0.913895	0.935555	756
PTBXL	PVC	0.90514	0.882495	0.923843	187
PTBXL	STTC	0.839615	0.811439	0.86978	181

**Table 4:** Comprehensive performance of the TimesNet model on the PTB-XL test dataset.

Class	AUC	Precision	Recall	F1-Score	Positive Samples
NORM	0.952	0.896	0.914	0.905	1412
AFIB	0.946	0.518	0.779	0.622	226
MI	0.924	0.720	0.831	0.771	756
PVC	0.905	0.412	0.652	0.505	187
STTC	0.840	0.290	0.591	0.389	181
Overall (Micro-Average)	0.913	0.700	0.841	0.764	—

**Table 5:** Performance of the TimesNet model evaluated using the Area Under the Receiver Operating Characteristic Curve (AUC) with corresponding 95% confidence intervals across PTB-XL training, validation, and test sets for NORM, AFIB, and PVC classes.

Split	Class	AUC	AUC_CI_Low	AUC_CI_High	Pos_Support
PTBXL_train	NORM	0.980789	0.977701	0.983573	6679
PTBXL_train	AFIB	0.966309	0.96148	0.971031	1033
PTBXL_train	PVC	0.937203	0.928956	0.944015	792
PTBXL_val	NORM	0.968723	0.957102	0.981024	1431
PTBXL_val	AFIB	0.953098	0.936275	0.967326	240
PTBXL_val	PVC	0.92458	0.903116	0.943646	165
PTBXL_test	NORM	0.976315	0.966769	0.983919	1404
PTBXL_test	AFIB	0.959554	0.946014	0.970867	241
PTBXL_test	PVC	0.930844	0.914221	0.94838	186

**Table 6:** Ablation analysis of the major architectural components in the proposed TimesNet-based ECG classification framework on the PTB-XL test set.

Configuration	FFT-Based Period Detection	Multi-Scale Times Blocks	Positional Embedding	Data Augmentation	Mean AUC
Full Proposed Model	Yes	Yes	Yes	Yes	0.913
Without FFT Period Extraction	No	Yes	Yes	Yes	0.900
Without Multi-Scale Kernels	Yes	No	Yes	Yes	0.892
Without Positional Embedding	Yes	Yes	No	Yes	0.904
Without Data Augmentation	Yes	Yes	Yes	No	0.898

**Table 7:** Computational complexity and scalability profile of the proposed periodicity-aware TimesNet framework.

Metric	Value
Model	Periodicity-aware TimesNet
Input ECG size	12 leads × 5000 samples
Trainable parameters	1.957 million
Computational complexity	13.14 GFLOPs/ECG recording
Average inference latency	5.230 ms/ECG recording
Median inference latency	5.229 ms/ECG recording
Latency standard deviation	0.064 ms
Throughput	191.21 ECG recordings/second
Peak allocated GPU memory	55.17 MB
Peak reserved GPU memory	76.00 MB
Hardware	CUDA GPU
Deployment implication	Indicates practical potential for scalable offline and cloud-assisted ECG analysis applications

**Table 8:** Comparison of the proposed framework with representative PTB-XL and ECG classification approaches.

Ref	Study/Model	Dataset/ECG Type	Classification Setting	Methodology	Key Contribution	Reported Metric	Performance
[22]	MTDL-Net (Han et al., 2023)	Multi-lead ECG	Multi-class	Transformer + temporal enhancement	Joint morphological and temporal feature learning	Accuracy, Recall	Acc: 95.6%, Recall: 96.4%
[24]	ML Arrhythmia Classification (Zakaria et al., 2024)	2-lead ECG	Arrhythmia classification	Ensemble ML + feature engineering	Inter-patient morphological classification	Accuracy, F1-score	Acc: 87%, F1: 87%
[25]	Morphology–Rhythm Contrast (Liu et al., 2024)	Multi-lead ECG	Multi-label	Triple-branch contrastive learning	Joint morphology–rhythm representation learning	AUROC, AUPRC	AUROC: 0.9889, AUPRC: 0.9694
[26]	MSGformer (Ji et al., 2024)	Multi-lead ECG	Multi-class	Multi-scale transformer + grid attention	Multi-scale spatial-temporal feature extraction	Accuracy, F1-score	Acc: 99.28%, F1 ≈ 0.86
[27]	1D-CNN + Attention (Guhdar et al., 2025)	Multi-lead ECG	Multi-class	CNN + attention + focal loss	Attention-based ECG representation learning	Accuracy, F1-score	Acc: 99.48–99.83%, F1 ≈ 1.00
[28]	Deep Learning ECG (Mathews et al., 2018)	Single-lead ECG	Beat classification	RBM + DBN	Early deep learning ECG classification	Accuracy	Acc: 93.63–95.57%
[29]	Y-Net-ECG (Liu et al., 2025)	Multi-lead ECG	AF detection	Dual-branch segmentation framework	Interpretable ECG segmentation and AF detection	F1-score, AUC	F1: 99.60%, AUC: 0.983
[30]	DeepMI (Tadesse et al., 2021)	12-lead ECG	MI classification	Multi-level fusion + RNN	Temporal myocardial infarction analysis	AUROC	AUROC: 96.7%
[31]	LVH Detection (Jothiramalingam et al., 2021)	Multi-lead ECG	Clinical diagnosis	ML + wavelet + classifiers	Clinical feature-based cardiac diagnosis	Accuracy	Acc: 97.8%
[32]	Multi-scale CNN (Zhou & Fang, 2025)	Multi-lead ECG	Multi-class	Hierarchical CNN + LEA	Multi-scale morphology-aware learning	Accuracy	Acc: 99.5%
[33]	Two-stage CNN (Jain et al., 2020)	Multi-lead ECG	Hypertension risk classification	CNN-based risk stratification	ECG-based hypertension risk assessment	Accuracy	Acc: 99.68%
[35]	ECG-MAE (Hu et al., 2023)	PTB-XL (12-lead ECG)	Multi-label	Self-supervised masked autoencoder	Label-efficient ECG representation learning	Macro-AUC	Macro-AUC: 0.9474
[34]	DDNN AF Detection (Cai et al., 2020)	12-lead ECG	AF classification	Dense convolutional neural network	Large-scale AF classification	Accuracy, Sensitivity	Acc: 99.35%, Sens: 99.19%
Proposed	Proposed TimesNet-Based Framework	PTB-XL (12-lead ECG)	Multi-label	FFT-based periodicity modeling + multi-scale TimesBlocks	Joint rhythm-aware and morphology-aware temporal representation learning	Mean AUC	Mean AUC: 0.96

## Data Availability

PTB-XL, a large publicly available electrocardiography dataset at https://physionet.org/content/ptb-xl/1.0.3/ (accessed on 14 November 2024).

## References

[1] Iqbal J.M. (2024). A novel deep learning approach for early detection of cardiovascular diseases from ECG signals. Med. Eng. Phys..

[2] Abubaker M.B., Babayiğit B. (2022). Detection of cardiovascular diseases in ECG images using machine learning and deep learning methods. IEEE Trans. Artif. Intell..

[3] Zeng W., Shan L., Yuan C., Du S. (2024). Advancing cardiac diagnostics: Exceptional accuracy in abnormal ECG signal classification with cascading deep learning and explainability analysis. Appl. Soft Comput..

[4] Sadr H., Salari A., Ashoobi M.T., Nazari M. (2024). Cardiovascular disease diagnosis: A holistic approach using the integration of machine learning and deep learning models. Eur. J. Med. Res..

[5] Hasan N.I., Bhattacharjee A. (2019). Deep learning approach to cardiovascular disease classification employing modified ECG signal from empirical mode decomposition. Biomed. Signal Process. Control.

[6] Alsayat A., Mahmoud A.A., Alanazi S., Mostafa A.M., Alshammari N., Alrowaily M.A., Shabana H., Ezz M. (2025). Enhancing cardiac diagnostics: A deep learning ensemble approach for precise ECG image classification. J. Big Data.

[7] Kalatehjari E., Hosseini M.M., Harimi A., Abolghasemi V. (2025). Advanced ensemble learning-based CNN-BiLSTM network for cardiovascular disease classification using ECG and PCG signal. Biomed. Signal Process. Control.

[8] Murat F., Yildirim O., Talo M., Baloglu U.B., Demir Y., Acharya U.R. (2020). Application of deep learning techniques for heartbeats detection using ECG signals-analysis and review. Comput. Biol. Med..

[9] Yu H., Lu Y., Zheng S. (2024). Inferring spatial–temporal dynamics of ECG signals with deep neural networks for cardiovascular diseases diagnosis. Biomed. Signal Process. Control.

[10] Kumar Behera S., Bhattacharya D., Aithal N., Sinha N. (2025). Non-linear dynamics in ECG: A novel approach for robust classification of cardiovascular disorders. npj Cardiovasc. Health.

[11] Yaser S., Chowdhury M.E., Sarmun R. (2025). SER inspired deep learning approach to detect cardiac arrhythmias in electrocardiogram signals using Temporal Convolutional Network and graph neural network. Comput. Biol. Med..

[12] Yu X., Ni H., Yan Z., Wang Z., Wang N. (2025). Interpretable Coronary Heart Disease Syndrome Differentiation and Identification Based on Pulse Signal. Biomed. Signal Process. Control.

[13] Kodipalli A., Rao T., Ushasree A., Sujatha C.N. (2026). Deep learning for electrocardiogram-based arrhythmia detection and classification: Architectures, challenges, and clinical translation. Deep Learning for Cardiac Signal Analysis in Robotic Applications.

[14] Qian L., Ellis H.L., Wang T., Wang J., Mitra R., Dobson R., Ibrahim Z. (2025). How deep is your guess? A fresh perspective on deep learning for medical time-series imputation. IEEE J. Biomed. Health Inform..

[15] Jia J., Jia A., Liu Z., Feng Y., Feng J. (2026). Atrial fibrillation detection method combining multi-scale bands and spatio-temporal features of ecg. Biomed. Signal Process. Control.

[16] Fan H., Luo J., Chang H., Han H., Tan J., Guo C., Li M., Wang Z. (2026). EQA-MDL: Wearable ECG Signal Quality Assessment via Multi-scale Difference Learning. IEEE J. Biomed. Health Inform..

[17] Wang C., Zhu M., Zhai S., Dawn B., Latifi S. Graph-Based Spatio-temporal Attention and Multi-Scale Fusion for Clinically Interpretable, High-Fidelity Fetal ECG Extraction. Proceedings of the 16th ACM International Conference on Bioinformatics, Computational Biology, and Health Informatics.

[18] Yang L., Wang C., Chu W., Chen H., Wu C., Chen Y., Wan X. (2026). Multiscale Feature Enhancement and Bidirectional Temporal Dependency Networks for Arrhythmia Classification. Biology.

[19] Xu D., Xu Y., Xu K., Hu Z., Xing M., Gini F., Greco M.S. (2025). WaveGRU-Net: Robust non-contact ECG reconstruction via MIMO millimeter-wave radar and multi-scale semantic analysis. Signal Process..

[20] Wu X., Yan M., Wang R., Xie L. (2025). Multiscale feature enhanced gating network for atrial fibrillation detection. Comput. Methods Programs Biomed..

[21] Wu H., Hu T., Liu Y., Zhou H., Wang J., Long M. (2022). Timesnet: Temporal 2d-variation modeling for general time series analysis. arXiv.

[22] Han C., Xiang S., Qian D. (2023). MTDL-NET: Morphological and Temporal Discriminative Learning for Heartbeat Classification. ICASSP 2023-2023 IEEE International Conference on Acoustics, Speech and Signal Processing (ICASSP).

[23] Doan Q.M., Dinh T.H., Singh A.K., Lin C.T., Trung N.L. (2024). Cascaded thinning in upscale and downscale representation for EEG signal processing. IEEE Trans. Neural Syst. Rehabil. Eng..

[24] Zakaria H., Nurdiniyah E.S.H., Kurniawati A.M., Naufal D., Sutisna N. (2024). Morphological arrhythmia classification based on inter-patient and two leads ecg using machine learning. IEEE Access.

[25] Liu W., Zhang H., Chang S., Wang H., He J., Huang Q. (2024). Learning representations for multilead electrocardiograms from morphology-rhythm contrast. IEEE Trans. Instrum. Meas..

[26] Ji C., Wang L., Qin J., Liu L., Han Y., Wang Z. (2024). MSGformer: A multi-scale grid transformer network for 12-lead ECG arrhythmia detection. Biomed. Signal Process. Control.

[27] Guhdar M., Mohammed A.O., Mstafa R.J. (2025). Advanced deep learning framework for ECG arrhythmia classification using 1D-CNN with attention mechanism. Knowl.-Based Syst..

[28] Mathews S.M., Kambhamettu C., Barner K.E. (2018). A novel application of deep learning for single-lead ECG classification. Comput. Biol. Med..

[29] Liu Y., Zhang P., Feng X., Hu D., Zhou D., Li J., Huang K., Zhao Y., Fu Z., Zheng Q. (2025). Y-Net-ECG: A Multi-Lead informed and interpretable architecture for ECG segmentation across diverse rhythms. Expert Syst. Appl..

[30] Tadesse G.A., Javed H., Weldemariam K., Liu Y., Liu J., Chen J., Zhu T. (2021). DeepMI: Deep multi-lead ECG fusion for identifying myocardial infarction and its occurrence-time. Artif. Intell. Med..

[31] Jothiramalingam R., Jude A., Patan R., Ramachandran M., Duraisamy J.H., Gandomi A.H. (2021). Machine learning-based left ventricular hypertrophy detection using multi-lead ECG signal. Neural Comput. Appl..

[32] Zhou F., Fang D. (2025). Classification of multi-lead ECG based on multiple scales and hierarchical feature convolutional neural networks. Sci. Rep..

[33] Jain P., Gajbhiye P., Tripathy R.K., Acharya U.R. (2020). A two-stage deep CNN architecture for the classification of low-risk and high-risk hypertension classes using multi-lead ECG signals. Inform. Med. Unlocked.

[34] Cai W., Chen Y., Guo J., Han B., Shi Y., Ji L., Wang J., Zhang G., Luo J. (2020). Accurate detection of atrial fibrillation from 12-lead ECG using deep neural network. Comput. Biol. Med..

[35] Hu R., Chen J., Zhou L. (2023). Spatiotemporal self-supervised representation learning from multi-lead ECG signals. Biomed. Signal Process. Control.

